# Aerobic exercise attenuates dysautonomia, cardiac diastolic dysfunctions, and hemodynamic overload in female mice with atherosclerosis

**DOI:** 10.1038/s41598-024-52883-x

**Published:** 2024-04-03

**Authors:** Bruno Nascimento-Carvalho, Bruno Durante da Silva, Maikon Barbosa da Silva, Adriano Dos-Santos, Thayna Fabiana Ribeiro, Danielle da Silva Dias, Leandro Eziquiel de Souza, Marina Rascio Henriques Dutra, Sergio Catanozi, Elia G. Caldini, Kátia De Angelis, Katia Bilhar Scapini, Iris Callado Sanches, Maria Claudia Irigoyen

**Affiliations:** 1 Unidade de Hipertensao, Instituto do Coracao, Hospital das Clinicas, Faculdade de Medicina, Universidade de Sao Paulo (InCor-HCFMUSP), São Paulo, SP Brazil; 2 Human Movement Lab, São Judas Tadeu University (USJT), São Paulo, SP Brazil; 3 Postgraduate Program in Physical Education, Universidade Federal Do Maranhão, São Luís, MA Brazil; 4 Laboratório de Fisiologia Translacional, Universidade Nove de Julho (Uninove), São Paulo, SP Brazil; 5 Physiology Exercise Laboratory, Department of Physiology, Federal University of São Paulo (Unifesp), São Paulo, SP Brazil; 6 Laboratorio de Lipides (LIM-10), Hospital das Clinicas (HCFMUSP), Faculdade de Medicina da Universidade de São Paulo, São Paulo, SP Brazil; 7 Departamento de Patologia, Faculdade de Medicina da Universidade de São Paulo, São Paulo, Brazil

**Keywords:** Cardiovascular diseases, Ageing, Atherosclerosis

## Abstract

Cardiovascular risk increases during the aging process in women with atherosclerosis and exercise training is a strategy for management of cardiac risks in at-risk populations. Therefore, the aims of this study were to evaluate: (1) the influence of the aging process on cardiac function, hemodynamics, cardiovascular autonomic modulation, and baroreflex sensitivity in females with atherosclerosis at the onset of reproductive senescence; and (2) the impact of exercise training on age-related dysfunctions in this model. Eighteen Apolipoprotein-E knockout female mice were divided equally into young (Y), middle-aged (MA), and trained middle-aged (MAT). Echocardiographic exams were performed to verify cardiac morphology and function. Cannulation for direct recording of blood pressure and heart rate, and analysis of cardiovascular autonomic modulation, baroreflex sensitivity were performed. The MA had lower cardiac diastolic function (E'/A' ratio), and higher aortic thickness, heart rate and mean arterial pressure, lower heart rate variability and baroreflex sensitivity compared with Y. There were no differences between Y and MAT in these parameters. Positive correlation coefficients were found between aortic wall thickness with hemodynamics data. The aging process causes a series of deleterious effects such as hemodynamic overload and dysautonomia in female with atherosclerosis. Exercise training was effective in mitigating aged-related dysfunctions.

## Introduction

Cardiovascular diseases persist as the primary cause of mortality globally, with coronary artery disease (CAD) ranking as the most lethal^1,2^. Atherosclerosis, a condition characterized by the buildup of plaque in the arteries, is a significant precursor to these diseases. When the coronary arteries are compromised, myocardial perfusion may be diminished or even obstructed, precipitating conditions like myocardial ischemia, angina, and in severe instances, myocardial infarction^3^.

The absence of apolipoprotein-E (ApoE) fosters hypercholesterolemia and spontaneous atherosclerosis in mice, mirroring the process observed in humans^4–6^. The progression of atherosclerotic plaques may lead to remodeling and damage to the aortic valve, inducing aortic regurgitation^4^, and cardiac diastolic dysfunction^6^. Accompanying these changes are an elevated expression of pro-inflammatory genes, an increased production of reactive oxygen species, and an escalated demand for the renin-angiotensin system. Collectively, these alterations stimulate sympathetic hyperactivation, which, over time, disrupts cardiovascular autonomic control and elevates blood pressure values^5^.

Although atheromatous plaques may be identified in younger individuals, the disease's progression is often intertwined with the aging process^7,8^. Moreover, previous research indicates that the aging process independently contributes to decreased running capacity, cardiac diastolic dysfunction with accompanying pathological cardiac hypertrophy, diminished baroreflex sensitivity (BRS) and an upsurge in inflammatory mediators in elderly control females^9,10^. Despite the cardioprotective influence of estrogen hormones before menopause^11^, an increase in the incidence of CAD has been documented in pre-menopausal women 35 years of age^12^. It is well established that the degenerative processes associated with aging are intensified in the presence of comorbidities like atherosclerosis^13^. Specifically, atherosclerosis triggers systemic inflammation, which may augment the detrimental effects of aging^14^. Therefore, we hypothesized that female mice with atherosclerosis may exhibit an exacerbation of the aging process effects on cardiovascular and autonomic outcomes, commencing from the onset of reproductive senescence.

Previous studies have demonstrated that exercise training (ET) may provide cardiovascular and autonomic benefits in young male mice^15^. In hypertensive female rats subjected to ovarian deprivation, ET has been found to enhance cardiac autonomic control and increase BRS^10,16^. Furthermore, ET has been shown to stabilize vascular injuries caused by atherosclerosis, along with a decrease in inflammation and oxidative stress in the mesenteric artery of Apolipoprotein-E knockout mice (ApoE-KO)^17,18^. Therefore, the additional hypothesis of this study suggests that ET might attenuate potential age-related detrimental effects on hemodynamic and autonomic outcomes in females with atherosclerosis at the beginning of reproductive senescence.

Consequently, this study examined two primary areas of interest (1) the influence of the aging process on cardiac function, hemodynamics, cardiovascular autonomic modulation and BRS in females with atherosclerosis at the onset of reproductive senescence; and (2) the impact of ET on age-related dysfunctions in this model.

## Methods

The experimental protocol was approved by the University of Sao Paulo Ethics Committee (protocol number 1297/2019), and this investigation was conducted following the Principles of Laboratory Animal Care formulated by the National Institutes of Health (National Institutes of Health Publication No., 96–23, Revised 1996). This manuscript was prepared according to the ARRIVE Guidelines Checklist for animal in vivo experiments. Experiments were performed on female ApoE-KO, from the Animal Care Facility of the Faculty of Medicine of the University of São Paulo, Brazil. The mice received standard laboratory chow and water ad libitum. They were housed in individual cages in a temperature-controlled room (22◦C) with a 12-h dark/light cycle.

### Study design

#### Animals

ApoE is a constituent of VLDL. It is synthesized in the liver and mediates high affinity to LDL receptors, being important for capturing lipoproteins in the bloodstream^4^. The ApoE-KO model was developed about three decades ago as the first murine model of atherosclerosis, through homologous recombination and inactivation of the ApoE gene^19^. Over time, the absence of ApoE promotes hypercholesterolemia and spontaneous atherosclerosis in mice, in a process very similar to what occurs in humans^4–6^. Thirty ApoE-KO mice were divided equally into three groups:

- Young group accompanied until the 6th month of life (Y)
- Middle-aged group accompanied until the 15th month of life (MA)
- Middle-aged training group trained in the last 6 weeks of the protocol and accompanied until the 15th month of life (MAT)

**Figure 1:**
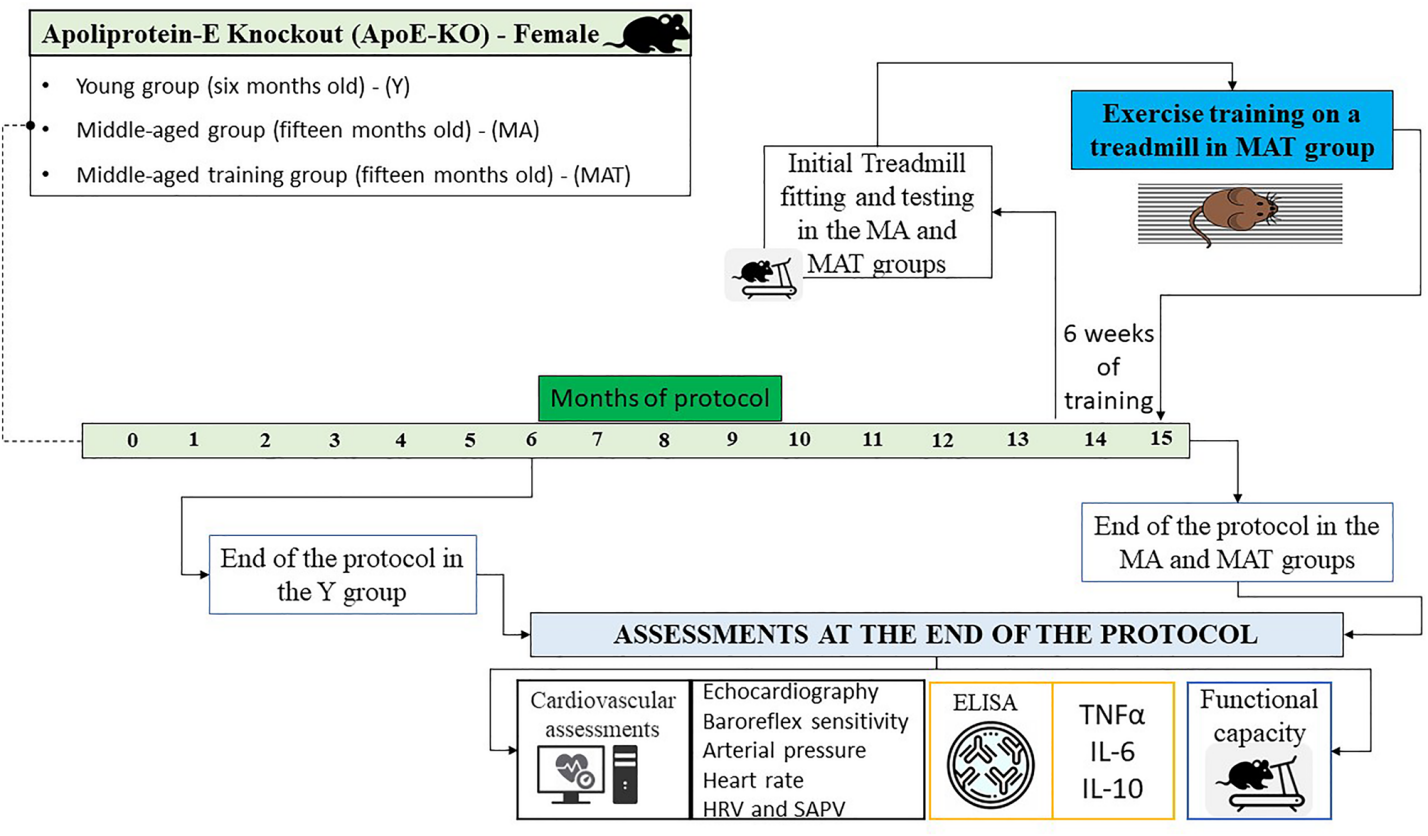
Protocol diagram.

To clarify the moments of the experiment, a protocol diagram is presented in Fig. 1.

### Aerobic exercise protocol

The animals were adapted to the maximal exercise test at the beginning of training (i.e., 0.3 km/h; 10 min/day/4 days). After 24 h, the animals underwent the progressive treadmill exercise test^15^. This test started with 0.3 km/h and increased progressively 0.3 km/h every 3 min until the animals reached a stage of exhaustion^15^. The MAT performed aerobic exercise training on a treadmill at moderate intensity (60–75% of the maximum treadmill test). The ET was performed during the last 6 weeks of the protocol (5 days/week, 1 h/day)^20^.

Additionally, all groups performed progressive exercise testing at the end of the protocol (the sedentary groups performed adaptation previously). The duration of the maximal exercise test was used for measuring the maximum running capacity of animals as an indicator of cardiovascular capacity^15,21^.

### Echocardiography analysis

Twenty-four hours after the last training session, echocardiography was performed in all groups, according to the guidelines of the American Society of Echocardiography. For this protocol, all animals were anesthetized (0.5–2% isoflurane), and images were obtained using a VEVO 2100 ultrasound machine (Visual Sonics, Toronto, Canada). Left ventricle dimensions and wall thickness were measured at the level of the papillary muscles in the left and right parasternal short axis during end-systole and end-diastole. Left ventricular ejection fraction (EF), mass, stroke volume, diastolic and systolic volume, heart rate (HR), and cardiac output were calculated^22^.

To characterize the diastolic function, maximum velocity of E and A waves were measured from the left ventricle filling flow, and maximum velocity of E’ and A’ waves from interventricular septum, isovolumic relaxation time (IVRT), and the E/A ratio, E’/A’ ratio, and E/E’ ratios were calculated. Global myocardial performance was calculated through the TEI index^15^. To estimate aortic wall thickness, external and internal diameters of the ascending aorta were measured, and the difference was divided by 2^23^.

### Hemodynamic measurements

Forty-eight hours after the last training session, the mice were anesthetized (mixture of 0.5%–2% isoflurane and 98% O_2_ at a flow rate of 1.5 L/min), and polyethylene tipped Tygon cannulas filled with heparinized saline were inserted into the carotid artery for direct measurements of arterial pressure (AP). Twenty-four hours after surgery, hemodynamic measurements were obtained, and the animals were awake and allowed to move freely in their cages. The cannula was coupled to a biological signal transducer for recording blood pressure signals (Blood Pressure XDCR, Kent Scientific, Litchfield, CT, USA) for 30 min using a digital converter (Windaq DI720, 4-kHz sampling frequency, Dataq Instruments) The recorded data were analyzed on a beat-to-beat basis to quantify changes in mean arterial pressure (MAP) and HR^24,25^.

### Heart rate variability

Pulse interval (PI) variability and systolic arterial pressure (SAP) variability were assessed in time and frequency domains by spectral analysis using Cardioseries Software (V2.7). For frequency domain analysis of cardiovascular autonomic modulation, PI and SAP were divided into segments and overlapped by 50%, cubic spline-decimated to be equally spaced in time after linear trend removal; power spectral density was obtained through the fast Fourier transformation. The components of spectral analysis were quantified in the very low-frequency ranges (VLF-PI, 0–0.4 Hz), low-frequency ranges (LF-PI, 0.4–1.5 Hz), and high-frequency ranges (HF-PI, 1.5–5.0 Hz)^25,26^.

### Baroreflex sensitivity evaluation

The α-index was obtained from the square root of the ratio between PI and SAP variability in the LF-PI^27^.

The baroreflex effectiveness index (BEI) and baroreflex gain index (BGI) were measured using Cardioseries Software (V2.7)^28^. The SAP ramps were determinate with three successive variances (up or down) of SAP. The baroreflex ramps were determined with three successive variances (up or down) of PI and SAP. The parameters for validating baroreflex sequence were PI and SAP threshold (0); delay (1); and minimum coefficient of correlation (r = 0.80). The BEI analyzed the ratio between the number of SAP ramps followed by the respective reflex PI ramps^29^. The BGI was used to compare the linear regression of SAP ramps with the that of the PI ramps^28^.

Moreover, tachycardic or bradycardic responses were induced by two injections of sodium nitroprusside (8 μg/kg body wt i.v.) or phenylephrine (8 μg/kg body wt i.v.), respectively^15,25^. Maximal dose per injection was < 20 µg^15,25^. Peak increases or decreases in MAP after phenylephrine or sodium nitroprusside injection and the corresponding peak reflex changes in HR were recorded for each drug dose. Data are expressed as beats per minute per mmHg (bpm/mmHg).

The drugs were administered randomly in all animals and response peaks were determined (maximum blood pressure change); 3- to 5-min intervals between doses were necessary for blood pressure to return to baseline. BRS was evaluated by a mean index relating changes in HR to the changes in MAP, allowing a separate analysis of gain for reflex bradycardia and reflex tachycardia^10^.

### Inflammatory mediators

One day after hemodynamic evaluations, the mice underwent euthanasia. Spleens, heart, and plasma were collected and immediately frozen at − 80 °C analyses. Interleukin 6 (IL-6), interleukin 10 (IL-10), and tumor necrosis factor alpha (TNF-α) levels were determined in the spleens by using a commercially available ELISA kit (R&D Systems Inc.), according to the manufacturer’s instructions. ELISA was performed in a 96-well polystyrene microplate with a specific monoclonal antibody coating. Absorbance was measured at 540 nm in a microplate reader^30^. Moreover, the ratio of pro-inflammatory to anti-inflammatory cytokines was performed to analyze the inflammatory profile^31^.

### Plasma nitrites

To determine the plasma nitrites (NO − _2_) we used the Griess reagent^32^.

### Cardiac stress oxidative

Cardiac oxidative stress analyzes were carried out in 6 animals from each group. Hydrogen peroxide concentration was assessed based on the horseradish peroxidase- (HRPO) mediated oxidation of phenol red by H2O2^32,33^. The activity of NADPH oxidase enzyme was determined by the production of superoxide determined by a plate reader^33^.

The antioxidant enzymes evaluated have already been previously described [Superoxide dismutase activity (SOD), Catalase activity and ferric reducing/antioxidant power (FRAP)^32,34^.

The lipid peroxidation was evaluated by thiobarbituric reactive substances (TBARS), and the determination of protein oxidation was determined by the carbonyl’s technique^32^.

### Collagen

Cardiac collagen analyzes were carried out in 4 animals from each group, sections were stained with picrosirius red for quantification of cardiac collagen. A total of nine or twelve fields per heart were analyzed with a 20 × objective magnification. Collagen fraction was determined by measuring the area of stained tissue within a given field and expressing that area as a proportion of the total area observed. Sections were stained with picrosirius red for quantification of cardiac collagen, the images were measured using the Image J^35^.

### Statistical analysis

The data were evaluated in Graph Pad Prism (V 8.0.1). The power statistic was analyzed in PSS Health^36^. The results are presented as mean ± SEM or median and interquartile range. Data homogeneity was tested using the Kolmogorov–Smirnov test. The experimental groups were compared using One-Way ANOVA with Tukey post hoc test, or non-parametric data were compared using Kruskal–Wallis with the Dunn post hoc test. The association between the variables was verified using the Pearson correlation. The significance level adopted was P < 0.05.

## Results

No differences were observed in the animals’ body weight (Y: 18.63 ± 0.38 g; MA: 20.50 ± 0.85 g; MAT: 20.40 ± 0.68 g; P = 0.07). Regarding running capacity at the baseline, no differences existed between MA and MAT (MA: 10.14 ± 0.31 m; MAT: 10.24 ± 0.53 m; P = 0.87). However, the MA had lower running capacity compared with the Y and MAT, whereas no differences were observed between Y and MAT at the end of protocol (Y: 13.36 ± 1.05 m; MA: 9.61 ± 0.56 m; MAT: 13.47 ± 1.18 m; P = 0.02).

**Figure 2:**
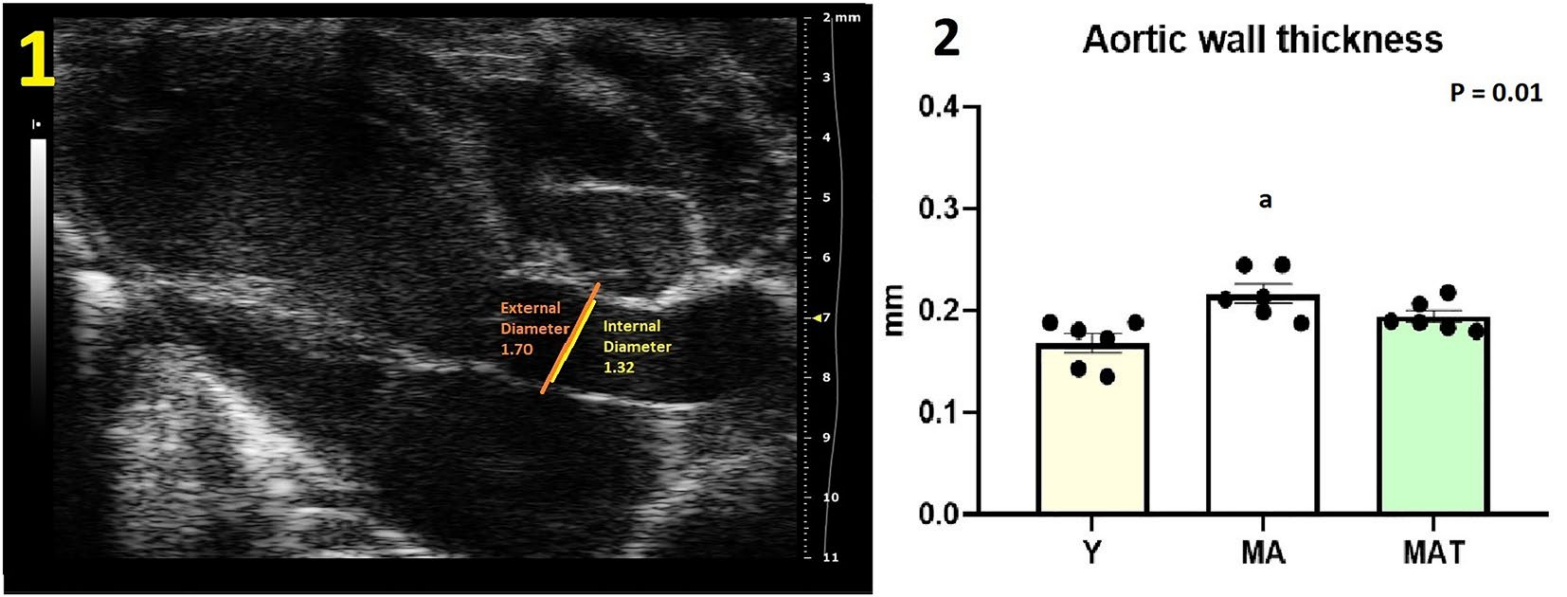
Aortic wall thickness assessments. Y, Young group (n = 6); MA, Middle-aged group (n = 6); MAT, Middle-aged trained (n = 6). 1 = Example of aortic wall thickness measurement; 2 = Aortic wall thickness. Symbol indicates statistically different groups (one-way ANOVA + Tukey test, P < 0.05). Values reported as mean ± SEM. ^a^P < 0.05 vs. Y.

The echocardiographic analyses are shown in Table 1. The MA had a lower E'/A' ratio compared with Y, indicating a worse cardiac diastolic function; however, no differences were noted between Y and MAT. In addition, aortic wall thickness was higher in MA compared with Y, whereas no differences occurred between Y and MAT (Fig. 2). No differences were observed in the other parameters.

**Table 1:** Echocardiography assessments.

Parameters	Y	MA	MAT	P	Power statistics (%)
Diastolic function					
E'/A'	2.17 (1.43–2.94)	0.70 (0.63–1.41)^a^	1.05 (0.79–1.32)	0.01	96.7
E/A	1.88 ± 0.35	1.07 ± 0.07	1.44 ± 0.17	0.07	66.6
E/E'	22.30 ± 2.46	27.16 ± 5.66	26.61 ± 0.62	0.61	34.8
IVRT (ms)	11.19 ± 2.19	17.72 ± 1.85	19.33 ± 3.01	0.06	74.7
Diastolic volume (uL)	64.90 ± 9.34	73.20 ± 7.81	67.90 ± 4.52	0.31	9.4
Systolic function					
Ejection fraction (%)	47.89 ± 5.62	48.69 ± 2.78	45.04 ± 1.84	0.78	9.1
Systolic volume (uL)	35.91 ± 7.60	37.63 ± 4.79	37.39 ± 2.90	0.97	5.4
Cardiac output (uL)	14,230 ± 1426	17,208 ± 2281	13,545 ± 815	0.27	29.6
Morphometry					
Left ventricular mass (mg)	68.95 ± 7.75	71.92 ± 4.40	75.99 ± 2.61	0.66	11.9
Global function					
MPI	0.89 ± 0.07	0.88 ± 0.06	0.88 ± 0.10	0.99	5.4

*Y* Young group (n = 6), *MA* Middle-aged group (n = 6), *MAT* Middle-aged trained (n = 6); Values reported as mean ± SEM or median and interquartile range. Different groups and type of test (parametric data compared one-way ANOVA + Tukey test or non-parametric data compared by using Kruskal–Wallis + Dunn test, P < 0.05).

^a^P < 0.05 vs. Y.

Hemodynamic parameters and cardiovascular autonomic modulation are reported in Table 2. Interestingly, we observed a higher SAP, diastolic arterial pressure (DAP) and MAP, as well as HR in the MA compared with the Y group, but all these parameters were similar between Y and MAT. Additionally, MA had lower heart rate variability (HRV) indexes, in the time and frequency domain compared with Y, and MAT had lower rates of cardiac parasympathetic modulation (RMSSD and HF-PI) in relation to Y (Y vs. MAT). In the meantime, there were no differences between Y and MAT in VLF-PI and LF-PI HRV indexes, and pulse interval variance (VAR-PI) was higher in MAT than in MA. Additionally, no differences were observed in the SAP variability values.

**Table 2:** Cardiovascular and autonomic assessments.

Parameters	Y	MA	MAT	P	Power statistics (%)
Hemodynamic					
DAP (mmHg)	89.99 (84.25–97.15)	100.70 (98.28–117.40)^a^	95.03 (93.76–97.16)	0.04	91.5
SAP (mmHg)	123.90 ± 4.96	153.80 ± 7.68^a^	143.50 ± 2.09	0.01	95.1
MAP (mmHg)	107.10 ± 3.23	130.4 ± 6.31^a^	119.8 ± 1.43	< 0.01	96.2
HR (bpm)	536 ± 40	664 ± 28^a^	613 ± 30	0.04	59.9
HRV—time domain					
VAR-PI (ms^2^)	15.44 ± 3.65	1.25 ± 0.10^a^	8.99 ± 2.06^b^	< 0.01	87.4
RMSSD (ms)	5.47 ± 1.03	0.99 ± 0.07^a^	1.51 ± 0.11^a^	< 0.01	100
HRV—Frequency domain					
VLF-PI (ms^2^)	4.66 (1.06–45.59)	0.15 (0.08–0.46)^a^	2.49 (1.31–4.59)	< 0.01	83.2
LF-PI (ms^2^)	1.31 (0.79–7.31)	0.07 (0.06–0.49)^a^	0.78 (0.30–0.95)	< 0.01	86.8
HF-PI (ms^2^)	8.21 ± 2.57	0.24 ± 0.03^a^	0.60 ± 0.08^a^	0.01	100
Systolic arterial pressure—time and frequency domain					
VAR-SAP (mmHg^2^)	14.21 ± 3.46	37.69 ± 9.41	26.89 ± 9.97	0.20	41.5
LF-SAP (mmHg^2^)	0.82 (0.35–3.71)	2.60 (1.40–7.77)	1.49 (0.68–3.42)	0.41	24

*Y* Young group (n = 6), *MA* Middle-aged group (n = 6), *MAT* Middle-aged trained (n = 6), *DAP* diastolic arterial pressure, *SAP* systolic arterial pressure, *MAP* mean arterial pressure, *HR* heart rate, *VAR-PI* variance of pulse interval, *RMSSD* root mean square of successive differences between normal heartbeats, *VLF-PI* very low-frequency of pulse interval, *LF-PI* low-frequency of pulse interval, *HF-PI* high-frequency of pulse interval, *VAR-SAP* variance of systolic arterial pressure, *LF-SAP* low-frequency of systolic arterial pressure. Values reported as mean ± SEM or median and interquartile range. Different groups and type of test (parametric data compared one-way ANOVA + Tukey test or non-parametric data compared using Kruskal–Wallis + Dunn test, P < 0.05).

^a^P < 0.05 vs. Y.

^b^P < 0.05 vs. MA.

MA had a lower α-index, and all indexes of BEI compared with Y (Y vs. MA), but no differences were observed between MAT and Y in these parameters. Additionally, MAT and MA had lower BGI up ramps than Y had (Table 3). Regarding the BRS data tested with vasoactive drugs, no differences were observed in the reflex bradycardic response. However, the tachycardic reflex response was lower in MA than in Y (Table 3).

**Table 3:** Baroreflex sensibility and inflammatory assessments.

Parameters	Y	MA	MAT	P	Power statistics (%)
α-index (LF-PI/LF-PAS)	1.48 ± 0.39	0.33 ± 0.05^a^	1.15 ± 0.30	0.04	80
BEI down (baroreflex ramps/SAP ramps)	0.14 ± 0.03	0.02 ± 0.01^a^	0.08 ± 0.01	0.01	97.70
BEI up (baroreflex ramps/SAP ramps)	0.15 ± 0.04	0.03 ± 0.01^a^	0.10 ± 0.02	0.04	82
BEI all (baroreflex ramps/SAP ramps)	0.14 ± 04	0.02 ± 0.01^a^	0.10 ± 0.02	0.02	93.7
BGI down (baroreflex ramps/SAP ramps)	3.66 ± 1.34	1.15 ± 1.21	1.02 ± 0.22	0.06	86.3
BGI up (baroreflex ramps/SAP ramps)	4.69 ± 1.46	0.99 ± 0.27^a^	1.03 ± 0.16^a^	0.01	97.7
BGI all (baroreflex ramps/SAP ramps)	4.19 ± 1.36	1.01 ± 0.28	1.02 ± 0.17	0.06	94.3
Tachycardic reflex (bpm/mmHg)	5.77 (4.18–7.30)	0.89 (0.79–2.41)^a^	3.09 (2.18–4.16)	< 0.01	98.4
Bradycardic reflex (bpm/mmHg)	− 5.03 (− 6.36– − 2.34)	− 2.18 (− 3.38– − 2.09)	− 4.89 (− 6.76– − 3.20)	0.17	39.9
TNF-α (pg/mg protein)	35.19 ± 4.62	43.00 ± 4.22	35.84 ± 1.85	0.27	63.9
IL-6 (pg/ mg protein)	23.22 ± 5.97	24.33 ± 1.17	25.04 ± 1.85	0.11	6.8
IL-10 (pg/ mg protein)	44.36 ± 6.92	26.86 ± 1.02^a^	30.32 ± 2.81	0.04	84
TNF-α/IL-10	0.79 ± 0.24	1.40 ± 0.36	1.23 ± 0.10	0.20	34
IL-6/IL-10	0.54 ± 0.13	0.91 ± 0.05^a^	0.90 ± 0.09^a^	0.02	91.5

*Y* Young group (n = 6); MA, Middle-aged group (n = 6), *MAT* Middle-aged trained (n = 6); Values reported as mean ± SEM or median and interquartile range. *BEI* baroreflex effectiveness index, *SAP* systolic arterial pressure, *BGI* baroreflex gain index. Different groups and type of test (parametric data compared with one-way ANOVA + Tukey test or non-parametric data compared using the Kruskal–Wallis + Dunn test, P < 0.05).

^a^P < 0.05 vs. Y.

The inflammatory analyses are shown in Table 3. MA had a lower IL-10 compared with Y (Y vs. MA), but no differences were observed between Y and MAT at the end of the protocol. Moreover, the IL-6/IL-10 ratio was higher in the middle-aged groups (Y vs. MA and MAT).

**Figure 3:**
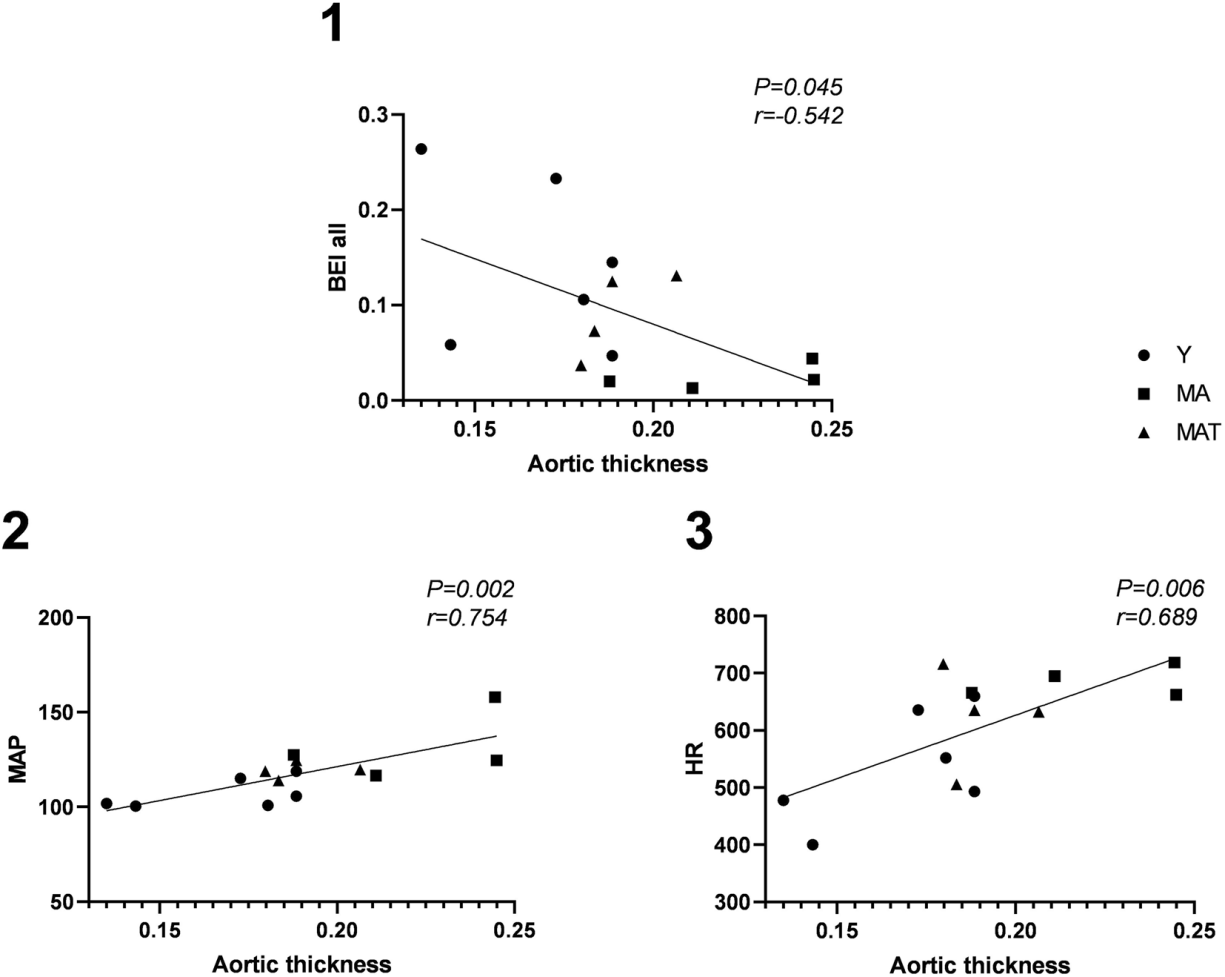
Correlations. 1 = Correlation between aortic wall thickness and baroreflex effectiveness index—all ramps; 2 = Correlation between aortic wall thickness and mean arterial pressure; 3 = Correlation between aortic wall thickness and heart rate.

Significant and strong positive correlation coefficients were found between aortic wall thickness and HR and aortic wall thickness and MAP. In addition, there was a negative correlation between aortic wall thickness and the all-ramps BEI (Fig. 3).

No differences were observed in the animals’ plasma nitrite (p = 0.32). Regarding cardiac stress oxidative data, MA had a lower SOD compared with Y (Y vs. MA) and no differences were observed in other parameters (Table 4).

**Table 4:** Cardiac oxidative stress assessments.

Parameters	Y	MA	MAT	P	Power statistics
Hydrogen peroxide (µM)	6.23 ± 0.48	6.49 ± 1.22	4.89 ± 0.75	0.50	26%
NADPH (nmol/mg protein)	0.13 ± 0.02	0.14 ± 0.03	0.11 ± 0.02	0.76	64.8%
SOD (USOD/mg protein)	9.35 ± 0.78	7.07 ± 0.63^**a**^	9.33 ± 0.54	0.04	81%
Catalase (nmol/mg protein)	0.46 ± 0.07	0.34 ± 0.04	0.36 ± 0.06	0.27	86.9
FRAP (µMFell/g)	0.31 ± 0.03	0.44 ± 0.08	0.35 ± 0.04	0.24	100%
Protein oxidation (μmol/mg protein)	2.35 ± 0.25	2.55 ± 0.31	2.67 ± 0.24	0.72	12.1%
Lipoperoxidation (μmol/mg protein)	32.31 ± 2.67	31.59 ± 4.50	25.57 ± 2.50	0.32	99.3%

*Y* Young group (n = 6), *MA* Middle-aged group (n = 6), *MAT* Middle-aged trained (n = 6); Values reported as mean ± SEM.

Different groups compared with one-way ANOVA, P < 0.05).

^a^P < 0.05 vs. Y.

**Figure 4:**
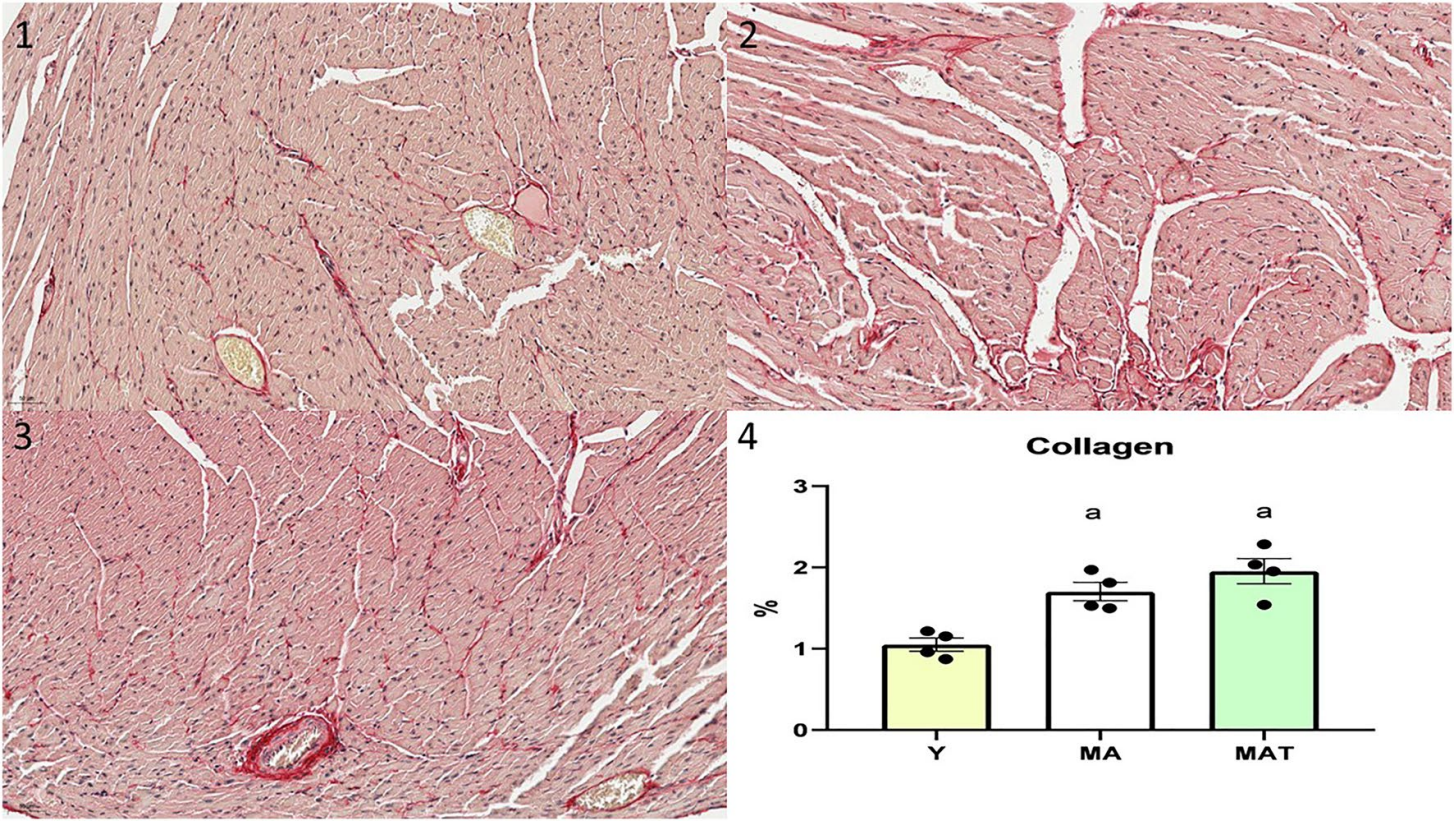
Cardiac collagen assessments. 1 = Young group histological image of the heart; 2 = Middle-aged group histological image of the heart; 3 = Middle-aged trained histological image of the heart; 4 = Cardiac collagen. Symbol indicates statistically different groups (one-way ANOVA + Tukey test, P < 0.05). Values reported as mean ± SEM. ^a^P < 0.05 vs. Y.

The collagen cardiac was higher in the middle-aged groups (Y vs. MA and MAT) (Fig. 4).

## Discussion

In the present study of female mice with atherosclerosis (ApoE-KO) at the beginning of reproductive senescence, the impact of the aging process on cardiac structure and function, hemodynamic parameters, and cardiovascular autonomic control were evaluated. Moreover, we also evaluated the effects of ET on aged-related dysfunctions. Our results demonstrated lower functional capacity, cardiac diastolic function, heart rate variability, and higher aortic wall thickness, arterial pressure and heart rate in MA compared with Y mice. However, the most important findings of the present study were that ET, only 6 weeks applied at the end of the protocol, mitigates aged-related alterations.

The reduction of functional capacity is a common characteristic resulting from the aging process in humans^37^ and in experimental models^9,10^. Therefore, the lower functional capacity in the MA group agreed with reports in the literature, but aerobic ET seems to reverse this decrease, and additionally, to increase the values of the maximum capacity test compared with Y in a condition like atherosclerosis, which is very relevant, considering that individuals with cardiovascular diseases have lower functional capacity and survival^38^. It is worth mentioning that there were no differences in the animals' body weight throughout the protocol.

The brain-derived neurotrophic factor (BDNF) is the major regulator of neural growth and one of the mechanisms that may justify the higher functional capacity observed in MAT^39,40^. Some genetic factors (myocyte-enhancer factor 2 [MEF2] and PGC-1α) interact with the brain through BDNF-dependent mechanisms and regulate several processes, such as muscle development and function, mitochondrial biogenesis, and lipid metabolism^40^, being determinant factors for physical performance. Furthermore, it has already been reported in the literature that associations may occur between functional capacity, increased serum levels of BDNF and cardiac autonomic modulation^39^.

Previous research demonstrated in analysis by strain that 100% of ApoE-KO animals have the presence of plaques showing that more than 50% of vessel area was constituted by atherosclerotic plaque area, in which a fibrous cap, cholesterol crystals, acellular regions, and lipid droplets were found at 8 months of age^41^. Considered that the collagen cardiac (senescence marker) and the aortic wall thickness were higher in middle-aged groups (Y vs. MA and MAT), and that this protocol observed the progression of atherosclerosis for 15 months, a period equivalent to the time when the production of estrogen hormones in female mice is reduced in the senescence process^13,42^, it is reasonable to postulate that the animals presented the natural changes of elderly in the atherosclerosis.

The reduction in cardiac diastolic function and changes in the structure of the left ventricle are hallmarks of the aging process^37^. In the present study, no differences were identified in the structure of the heart in the echocardiography, but the aging increased cardiac collagen in middle-aged groups (MA and MAT), and a lower E'/A' ratio was observed in MA compared with Y, which indicates a lower cardiac diastolic function. Consistent evidence demonstrates increase in myocardial collagen associated with aging, and the accumulation of collagen may alter myocyte loading, leading to cardiac hypertrophy, as well as may promoting an increase in stiffness during diastole^43^. However, the trained group (MAT) has a greater amount of collagen and did not present diastolic dysfunction.

It is already known that impairment of diastolic function in mice may be related with reduced aortic valve function, indeed, in this study the animal with diastolic disfunction are the same animals that presented greater aortic wall thickness^6^. It is important to mention that there is a high incidence of aortic regurgitation in ApoE-KO animals experiencing the aging process^4^ and previous research indicates that this process in the ApoE-KO may provide increased aortic stiffness with higher pulse wave velocity and peak aortic flow^44^.

Considering that oxidative stress and inflammation are mechanisms that act in an interrelated way, generating a feedback loop for the progression of atheroma plaques and extracellular matrix degradation^45^, and that these mechanisms may induce the lipid infiltration in mice aorta^46^, suggests that the advancement of the aging process may be associated with these changes. In fact, increasing the amount of vascular lipid infiltrates, impairing arterial compliance aortic, leading to cardiac diastolic dysfunction in MA group may be linked with the reduction in antioxidant defense (SOD) associated with the pro-inflammatory profile (higher IL-6/IL-10 ratio and lower IL-10) and the greater thickness of the aortic wall in the MA group, in the present study.

In male ApoE-KO mice that performed the aerobic training protocol of 15/16 weeks duration at moderate intensity, there was a reduction in atherosclerotic content associated with endothelial benefits, oxidative stress, and inflammatory profile^18^. It also promotes stabilization of the atherosclerotic plaque with maintenance of the lipid profile, with a 6-week protocol also at moderate intensity^17^. These pieces of evidence suggest that the influence of aerobic ET on controlling the progression of atherosclerotic plaques is dependent on the number of atherosclerotic plaques established, as well as the training volume.

SAP, DAP, and MAP, as well as HR were higher in MA than in Y mice. Indeed, the systemic increase in blood pressure in elderly female rats has been previously reported in the literature^9^. This study demonstrates the influence of atherosclerosis in this context. The lower vascular responsiveness previously identified in this model of aging^4^, the constriction of the vessel commonly demonstrated with the progression of atherosclerosis, and lower baroreceptor efficiency identified in this study may justify the higher blood pressure values. It is worth mentioning that the deficit of ApoE generates a series of organic changes, such as imbalance in cholesterol metabolism, systemic inflammation, increased production of reactive oxygen species, reduced expression of antioxidant genes, increased demand for renin-angiotensin system and degradation of the extracellular matrix^5^ which may have contributed to the process of dysautonomia and vascular damage.

Previous studies have demonstrated that Angiotensin II may accelerates the development of atherosclerosis in ApoE-KO^47,48^, increasing atherosclerotic lesions with a vulnerable phenotype by activation of MMP8 and MMP13^49^ and forming aneurysms in the aorta, without changing hemodynamics^47^ Therefore, it is important to investigate the impacts of aging and exercise training on level of Angiotensin II and the cardiovascular repercussions on Apoe-KO female in future studies.

Several cardiology societies recommend ET as an efficient non-pharmacological strategy to control/manage high blood pressure^50^. In addition, studies demonstrate that aerobic training reduces blood pressure values in females with risk factors, such as the absence of ovarian hormones and hypertension^16,51^. Although this study does not present vascular histology, the similar thickness of the aorta between MAT and Y mice and the positive correlation between aortic wall thickness and MAP suggest that ET may have promoted vascular adaptations that contributed to a systemic reduction in blood pressure values.

The aortic arch is the anatomical location of one set of arterial stretch baroreceptors; therefore, it is reasonable to postulate that stiffness of the aortic wall modulates in vivo baroreceptor function^52^. The association of aortic wall thickness with baroreflex sensitivity, HR and MAP reinforces the concept that changes in this structure may compromise baroreceptor sensitivity and, subsequently, hemodynamic balance. ET may stabilize vascular injuries resulting from atherosclerosis independently of changes in plasmatic lipids^17^. Furthermore, it has already been shown that exercise training may attenuate endothelial dysfunction, inflammation, and oxidative stress in the mesenteric artery^18^. Additionally, arterial stiffness decreased significantly after moderate-intensity aerobic exercises in middle-aged women^53^.

Aerobic exercise training seems to attenuate the hemodynamic overload, the higher aortic wall thickness and the lower baroreflex sensitivity, consider that, no differences were observed between MAT and Y mice. One hypothesis for these results is that the exercise training may attenuate the decline in antioxidant (SOD) and anti-inflammatory (IL-10) mechanisms, reducing lipid adhesion and consequently attenuating the increase in aortic thickness, factors that improve aortic compliance and stretch capacity, and consequently, optimize the performance of baroreceptors. This improvement of short-term blood pressure mechanism regulation is probably associated with a reduction greater than 10 mmHg in MAP and SAP in MAT when comparing with MA. Although the values were not statistically different this decrease is relevant from a clinical point of view, considering that for each 5-mmHg reduction in systolic blood pressure, the risk of developing cardiovascular events lower by 10%^54^.

Resting bradycardia is a classic finding in trained individuals. Indeed, aerobic training promotes a constant increase in venous return and systolic volume, which constantly trigger the Frank-Starling mechanism and modify the cardiac contractility pattern, enhancing the effectiveness of the cardiac muscle^55^. ET has been demonstrated to increase the HRV in female rats^9,10^. In this study, the MAT mice did not have resting bradycardia (vs. MA). However, the MAT had similar values to Y in HR, E'/A' ratio, and HRV indexes (VLF-PI; LF-PI); moreover, the MAT had higher VAR-PI than MA mice, demonstrating that the ET may promote cardiac autonomic modulation benefits in female mice with atherosclerosis at the onset of reproductive senescence.

The imbalance in cardiovascular autonomic control associated with reduced BRS is a finding that has already been demonstrated in older female rats^9,10^. Therefore, considering the additional risks promoted by the progression of atherosclerosis, lower cardiovascular autonomic control was expected in the MA. In fact, the HRV data, both in the time and frequency domains, showed a drop in cardiac autonomic modulation in the MA compared with the Y mice, indicating dysautonomia in senescent females with atherosclerosis.

Although MA had a lower LF-PI band (parameter with a predominance of cardiac sympathetic modulation), the results of HRV should be analyzed together because lower values were observed in all parameters, indicating that the autonomic nervous system of these animals has a lower ability to modulate the heartbeat independently of the predominance of the sympathetic/parasympathetic loop. The lower tachycardic reflex in MA demonstrates an inability to adapt to pressure variations, which may have been generated by a reduction in the sensitivity of the baroreceptors caused by the progression of atherosclerosis. This process may be linked to the damage to afferent fibers, as well as to the reduction of the autonomic nervous system’s ability to quickly adapt to stimuli due to a long period of sympathetic hyperactivity.

The negative correlation between aortic wall thickness and spontaneous BRS does not concretely indicate that changes in the aortic wall thickness are a determining mechanism for modifying the sensitivity of arterial baroreceptors; however, the data demonstrate an association between the progression or stabilization of atherosclerotic disease with the cardiovascular autonomic control in females with atherosclerosis. Some mechanisms already described in the literature may explain the process of dysautonomia observed in ApoE-KO animals. There may be a reduction in the efficiency of arterial baroreceptors, a result of systemic damage in their afferences, due to increased production of pro-inflammatory cytokines and activation of Ang II, degradation of the extracellular matrix, deposit of lipids and establishment of atherosclerosis^5^. Besides the lower baroreflex efficiency, there may be a reduction in oxidant defense with an increase in reactive oxygen species^45^ and reduced bioavailability of nitric oxide, generating sympathetic nervous hyperactivation^5^.

Although the systemic inflammation is a classic finding that occurs with the progression of atherosclerosis ^14^ and studies demonstrated that the inflammatory changes in the spleen are similar to the alterations occurring in the cardiovascular system of rats^56^. Aging was expected to promotes the increase in the IL-6/IL-10 ratio, reduction in anti-inflammatory cytokines (IL-10) and reduce antioxidant defense (SOD). With the advancement of atherosclerosis in aging, oxidative stress (increasing oxidants in relation to antioxidant defense) and systemic inflammation in ApoE-KO animals occur^45^. However, it is essential to mention that ET may reestablish IL-10 and the training also attenuated the lower autonomic modulation. One hypothesis for this finding is that the cholinergic anti-inflammatory reflex was activated by the autonomic nervous system control ^57^. But the higher IL-6/IL-10 ratio in the trained group suggests a sustained systemic inflammation, indicating that the cardiovascular benefits promoted by training were determined predominantly by other mechanisms, such as autonomic control. The lower BRS identified in the MA reinforces the dysautonomia and demonstrates a lower ability of adaptation to pressure variations. These data are extremely worrisome because individuals with impaired baroreflex and lower HRV have a lower survival rate^58^.

ET has the capacity to promote improvements in BRS and a better prognosis in patients, even in extreme conditions, such as post-acute myocardial infarction^59^. It is also known that ET may potentially promote changes in mice's autonomic cardiovascular balance ^15^. In the present study, the ET seems to have promoted a series of adjustments, such as preventing the increase in aortic wall thickness, and restoring cardiac autonomic modulation (VAR-PI) and BRS. These changes indicate that this strategy promoted better control over the progression of atherosclerosis and was effective in restoring cardiovascular autonomic control and the action of baroreceptors.

In conclusion, aging promoted cardiac diastolic, hemodynamic and heart rate variability dysfunctions, as well as increased aortic wall thickness in females with atherosclerosis at the beginning of reproductive senescence. Importantly, ET appears to be effective in mitigating aged-related impairments in structural and functional parameters; therefore, it is a strategy for promoting autonomic and cardiovascular benefits in senescent females with atherosclerosis.

## Data Availability

The data that support the findings of this study are available from the corresponding author, MCI, upon reasonable request.
